# Current situation and influencing factors of oral frailty among community-dwelling older adults with chronic diseases in China: a cross-sectional study

**DOI:** 10.3389/fpubh.2026.1838310

**Published:** 2026-10-02

**Authors:** Yuan Zhou, Gui-Meng Wang, Ji-Sook Kang

**Affiliations:** 1 College of Nursing, Wonkwang University, Iksan, Jeonbuk Province, Republic of Korea; 2 School of Health Policy & Management, Nanjing Medical University, Nanjing, China; 3 School of Nursing, Yanbian University, Yanji, Jilin Province, China

**Keywords:** chronic diseases, community health services, cross-sectional study, multimorbidity, older adults, oral frailty

## Abstract

**Objective:**

To examine the prevalence and associated factors of oral frailty among community-dwelling older adults with chronic diseases in minority communities in Northeast China.

**Methods:**

A cross-sectional study was conducted from November to December 2023 using convenience sampling in three communities in Yanji City, Yanbian Korean Autonomous Prefecture. Participants aged 60 years and older with at least one diagnosed chronic disease and a minimum of four natural teeth completed the Mandarin Chinese versions of the Oral Frailty Index-8 (OFI-8), the FRAIL scale, the Pittsburgh Sleep Quality Index (PSQI), and the Attitudes to Aging Questionnaire (AAQ). Binary logistic regression was used to identify factors independently associated with oral frailty.

**Results:**

Among 393 valid participants, oral frailty was identified in 280 individuals, yielding a prevalence of 71.2% (OFI-8 score: 5.61 ± 2.58). The most frequently reported OFI-8 items were difficulty eating hard food (62.3%), choking (48.6%), and dry mouth (45.8%). After adjustment, advanced age, female sex, low personal monthly income, both low and high BMI, alcohol consumption, physical frailty, sleep disturbance, a negative attitude toward aging, having three or more chronic diseases, and diabetes mellitus were independently associated with oral frailty (all *p* < 0.05).

**Conclusion:**

Oral frailty is highly prevalent among older adults with chronic diseases in minority communities of Northeast China and is independently associated with multimorbidity, particularly diabetes. Screening for oral frailty should be integrated into routine community-based chronic disease management, with targeted prevention efforts focusing on modifiable behavioral and psychosocial determinants.

## Introduction

The world is experiencing an unprecedented demographic transition. By 2050, the World Health Organization projects that approximately 434 million people will be aged 80 years or older, with more than 60% of those aged 65 years and above residing in Asia. As populations age, chronic non-communicable diseases have supplanted infectious illnesses as the primary public health challenge: approximately 81% of older Chinese adults live with at least one chronic disease, and more than half have two or more. Consequently, the clinical and economic burden of multimorbidity—and the associated frailty syndromes—has become a defining priority for global health systems (1–6). Within the broader frailty framework, oral frailty has recently emerged as a distinct concept. First proposed in Japan by Tanaka and colleagues, oral frailty is defined as the age-related, multidimensional decline of orofacial structures and functions, encompassing the loss of natural teeth, reduced masticatory and swallowing performance, articulatory decline, salivary hypofunction, and diminished interest in oral self-care, accompanied by concurrent declines in physical, cognitive, and social functions (7–9). The recent concept analysis by Yang et al., using the Walker and Avant method, has further clarified its attributes (abnormal oral structure and/or multifaceted oral functional decline), antecedents (aging, social frailty, severe periodontitis), and consequences (poor nutrition, sarcopenia, cognitive decline, social withdrawal, mortality) (10).

It is important to distinguish oral frailty from related but conceptually narrower constructs. Oral hypofunction, formally classified as a disease under the 2018 Japanese National Health Insurance system, is diagnosed when three or more of seven measurable signs are present: poor oral hygiene, oral dryness, reduced occlusal force, decreased tongue–lip motor function, decreased tongue pressure, decreased masticatory function, and deterioration of swallowing function. Most of these signs require specialized dental equipment for assessment. Oral hypofunction is best regarded as a core component nested within the broader, more comprehensive construct of oral frailty (11, 12). In contrast, single oral health indicators, such as remaining tooth count, periodontal status, or salivary flow-capture only one dimension of oral health and lack the predictive value of the integrated oral frailty construct. Critically, oral frailty is reversible at its early stage, making it a uniquely valuable target for community-based screening and prevention. It is operationalized through brief self-report instruments, such as the eight-item Oral Frailty Index (OFI-8), which can be administered without specialized equipment. These features make oral frailty, rather than any single oral health parameter, the most suitable construct for public health surveillance in vulnerable populations.

Several biological mechanisms have been proposed to drive oral frailty. First, age-related sarcopenia of the orofacial musculature—including the masseter, temporalis, suprahyoid, and oropharyngeal muscles—reduces bite force, tongue pressure, and swallowing performance. Second, salivary hypofunction and oral microbial dysbiosis predispose individuals to caries, periodontitis, and oral candidiasis. Third, neuromuscular decline affecting motor coordination compromises chewing rhythm and bolus formation. Fourth, chronic low-grade systemic inflammation creates a bidirectional link between oral and systemic aging (13, 14). Through these mechanisms, oral frailty has been shown to predict malnutrition, sarcopenia, cognitive impairment, physical frailty, depression, and all-cause mortality (15–17).

The specific significance of oral frailty in older adults with chronic diseases warrants particular emphasis. First, older adults with chronic conditions—especially diabetes mellitus—exhibit significantly lower skeletal muscle mass and a higher chronic inflammatory burden than their healthy peers, both of which directly compromise orofacial musculature (18, 19). Second, polypharmacy is common in this population: more than 60% of community-dwelling older adults with chronic diseases take five or more medications daily, many of which (including antihypertensives, antidepressants, antihistamines, diuretics, opioids, and anticholinergics) cause xerostomia, predisposing them to caries, periodontitis, candidiasis, and impaired mastication and swallowing (20). Third, diabetes mellitus, in particular, has a direct and bidirectional impact on oral health: hyperglycemia accelerates periodontal disease, impairs wound healing, increases salivary glucose concentrations, and promotes oral microbial dysbiosis (21–23). A recent systematic review and meta-analysis by Wang et al. confirmed that older adults with type 2 diabetes have a markedly elevated prevalence of oral frailty compared with non-diabetic peers, with age, diabetes duration, polypharmacy, and HbA1c emerging as key determinants (24, 25). Fourth, cardiovascular disease, chronic respiratory disease, and chronic kidney disease share inflammatory and vascular pathways that further accelerate orofacial decline. Despite this strong rationale, evidence on oral frailty specifically in older adults with chronic diseases—and on the dose–response relationship between multimorbidity and oral frailty—remains limited. Existing studies have largely focused on general community-dwelling older populations rather than this high-risk subgroup.

China, the largest developing country, is experiencing rapid demographic aging; however, community-based oral healthcare services remain underdeveloped, particularly in the northeastern regions where economic development lags behind that of the coastal provinces. Yanji City, located in Jilin Province within the Yanbian Korean Autonomous Prefecture, is a minority-ethnic region characterized by comparatively lower educational attainment and socioeconomic status (26). Traditional Korean dietary patterns, which are rich in high-salt fermented foods, combined with the burden of chronic diseases, may further exacerbate disparities in oral health within this population. Therefore, examining the prevalence and determinants of oral frailty in older adults with chronic diseases in this specific context—and explicitly evaluating multimorbidity and disease-specific factors—is a crucial step toward developing equitable healthy aging strategies.

Accordingly, this cross-sectional study aims to (i) document the prevalence and item-level distribution of oral frailty among community-dwelling older adults with chronic diseases in minority communities of Northeast China; (ii) identify demographic, behavioral, physical, and psychosocial factors independently associated with oral frailty; and (iii) explicitly evaluate the contribution of the number of chronic diseases and diabetes mellitus to oral frailty risk in this population. The findings are expected to provide an empirical basis for developing a risk prediction model and designing targeted early intervention strategies that integrate oral health into routine community-based chronic disease management.

## Materials and methods

### Study design

A cross-sectional study was conducted between November and December 2023 and reported in accordance with the Strengthening the Reporting of Observational Studies in Epidemiology (STROBE) statement (27).

### Setting and sample

Participants were recruited from three communities in Yanji City, Jilin Province, Northeast China, using convenience sampling. Eligibility criteria were: (1) age ≥60 years; (2) residence in the selected community for ≥6 months; (3) at least one chronic disease diagnosed by a Grade A tertiary or higher hospital under the national hospital-classification system; (4) a minimum of four natural teeth excluding third molars; (5) clear consciousness, adequate communication ability, and sufficient comprehension to complete the questionnaire independently or with researcher assistance; and (6) written informed consent. Participants were excluded if they had severe cognitive impairment or psychiatric disorders, significant organ dysfunction (e.g., heart, brain, or kidney failure), or were in an acute phase of illness.

Rationale for the four-natural-teeth criterion. A minimum of four natural teeth (excluding third molars) was required for participation for three reasons. First, several OFI-8 items (e.g., difficulty eating hard food, choking, dry mouth, articulation) presuppose a minimum amount of remaining dentition for a meaningful response; respondents with near-total tooth loss who wear complete dentures would systematically endorse the chewing/articulation items in ways that do not reflect age-related oral functional decline. Second, this criterion has been used in previous Chinese community studies of oral frailty (Yin et al., 2024; Wang Lin et al., 2023), facilitating direct prevalence comparison. Third, oral frailty is conceptually distinct from edentulism; participants with near-total tooth loss represent an end-stage state for which separate screening instruments are recommended (28). In our study only 12 of the 421 initially screened individuals (2.8%) were excluded on this criterion.

Sample size. The required sample size was calculated using the multivariate-analysis principle that the sample should be 10–15 times the number of independent variables (29). With 18 variables and the upper limit (×15), a minimum of 270 participants was required; allowing for a 20% rate of invalid questionnaires, the final calculated minimum was 324. We ultimately recruited 409 older adults, of whom 393 (96.1%) provided valid questionnaires for analysis.

### Ethics

The study was approved by the Medical Ethics Committee of Yanbian University (20231107) and was conducted in accordance with the Declaration of Helsinki. All participants provided written informed consent.

### Measurements

Sociodemographic and clinical questionnaire. A structured questionnaire collected information on gender, age, ethnicity, residential location, monthly income, educational level, family structure, type of health insurance, living arrangements, and marital status. Health-related variables were also assessed: smoking (continuous or cumulative tobacco use for ≥6 months); alcohol consumption (occasional, regular or daily); regular physical exercise (≥3 sessions per week, ≥30 min/session, sustained for ≥6 months); body-mass index (BMI = weight [kg] / height^2^ [m^2^]) categorized as underweight (<18.5), normal (18.5–23.9), overweight (24.0–27.9), mild obesity (28.0–29.9) and moderate obesity (30.0–34.9) following the Chinese reference (30); self-rated health; and presence of physical disability. Chronic-disease assessment. The number and types of chronic diseases were ascertained according to the International Classification of Diseases, 10th Revision (ICD-10) (31) and verified against participants’ medical records or prescription documentation where possible. Diseases assessed individually included hypertension, type 2 diabetes mellitus, coronary heart disease, cerebrovascular disease (stroke/TIA), chronic respiratory disease (COPD, chronic bronchitis, asthma), osteoarthritis/osteoporosis, chronic kidney disease, malignant tumor, and others. The number of co-occurring chronic diseases was categorized as 1, 2, and ≥3 (multimorbidity).

*Oral Frailty Index-8 (OFI-8).* The Mandarin Chinese version of the OFI-8 (32, 33), originally developed by Tanaka et al. (9) was used. The instrument consists of eight items with a weighted scoring system: items 1–3 score 2 points for “yes” and 0 for “no”; items 4–5 score 1 for “yes” and 0 for “no”; items 6–8 are reverse-scored. The total score ranges from 0 to 11, with 0–2 indicating low risk, 3 representing moderate risk, and ≥4 high risk of oral frailty. The cut-off of ≥4 has been established to yield ~80% sensitivity and ~80% specificity for oral frailty and has been validated cross-culturally in Mandarin Chinese, Korean and Portuguese populations (33–35). The internal consistency in our sample was acceptable (Cronbach’s *α* = 0.702).

*FRAIL scale.* The Chinese version of the FRAIL scale (Wei et al.) (36) was used to assess physical frailty. The five components (fatigue, resistance, ambulation, illness, loss of weight) are each scored 0/1, and a total score ≥3 indicates physical frailty. Cronbach’s *α* in our sample = 0.706.

*Pittsburgh Sleep Quality Index (PSQI).* The Chinese version (Liu et al.) (37) was administered. The 18-item PSQI yields seven component scores summing to a global score of 0–21; a global score >7 indicates sleep disturbance. Cronbach’s *α* in our sample = 0.719.

*Attitudes to Aging Questionnaire (AAQ).* The Chinese version (Huang et al.) (38) was used. The 24 items rated on a 5-point Likert scale yield a total score of 24–120; a score ≥72 indicates a positive aging attitude. Cronbach’s *α* in our sample = 0.834.

### Data collection

Five graduate nursing students received standardized training before data collection to ensure consistency and methodological rigor. Eligible older adults were invited to participate and paper-based questionnaires were administered on site. Investigators offered individual guidance when needed; for participants with low literacy or visual impairment a structured-interview format was adopted, in which investigators read each question aloud and recorded the responses. Completed questionnaires were checked immediately for missing/inconsistent responses, which were clarified on the spot. Data were stored securely with access limited to authorized research team members. Double data entry was used to minimize input error. Of 409 distributed questionnaires, 393 were valid (96.1%).

### Statistical analysis

All analyses were performed with SPSS version 28.0 (IBM Corp., Armonk, NY, USA). Continuous variables were checked for normality with the Shapiro–Wilk test and visual inspection; normally distributed variables are presented as mean ± standard deviation and compared with the independent-sample t-test (two-group) or one-way ANOVA (>two groups). Categorical variables are presented as frequency (%) and compared with the chi-square test or Fisher’s exact test where expected cell counts were small. Ordinal variables (e.g., BMI category, income category, number of chronic diseases) were treated as categorical and dummy-coded for regression analysis. Because oral frailty was modeled as a binary outcome, binary logistic regression was used to identify independent associations. Variables were entered into the multivariable model if they met at least one of the following criteria: (a) *p* < 0.10 in the between-group comparison; or (b) prior evidence of clinical or theoretical importance. Under criterion (b), educational level, number of chronic diseases and diabetes mellitus were retained regardless of univariate *p*-value. Multicollinearity was assessed using variance inflation factors (VIF), all of which were < 2.5. Model fit was evaluated using the omnibus test of model coefficients, the Hosmer–Lemeshow goodness-of-fit test, and Nagelkerke R^2^. Two-tailed *p* < 0.05 was considered statistically significant.

## Results

### Participant characteristics

A total of 393 community-dwelling older adults with chronic diseases were included in the final analysis. The mean age was 68.7 ± 6.0 years; 162 (41.2%) were male and 231 (58.8%) female. Ethnic composition was 216 (55.0%) Han Chinese and 177 (45.0%) ethnic Korean Chinese. Most participants lived with a spouse (*n* = 314, 79.9%). The mean FRAIL score was 2.52 ± 0.87, with 195 participants (49.6%) meeting criteria for physical frailty. The mean PSQI score was 7.12 ± 3.26, with 155 (39.4%) experiencing sleep disturbance. The mean AAQ score was 69.3 ± 9.8, with 250 (63.6%) holding a negative attitude toward aging. Demographic, behavioral, physical and psychosocial characteristics of participants with and without oral frailty are shown in Table 1.

**Table 1:** Comparison of demographic, behavioral, physical and psychosocial characteristics between participants with and without oral frailty (*n* = 393).

Variable	No oral frailty (*n* = 113)	Oral frailty (*n* = 280)	Test statistic	*p*-value
Sex, n (%)			5.21	0.022
Male	59 (52.2)	103 (36.8)		
Female	54 (47.8)	177 (63.2)		
Age group (years), n (%)			12.45	0.002
60–69	84 (74.3)	168 (60.0)		
70–79	27 (23.9)	91 (32.5)		
≥80	2 (1.8)	21 (7.5)		
Ethnicity, n (%)			15.84	<0.001
Han Chinese	41 (36.3)	175 (62.5)		
Korean Chinese	72 (63.7)	105 (37.5)		
Personal monthly income (RMB), n (%)			58.42	<0.001
None	2 (1.8)	20 (7.1)		
<1,000	2 (1.8)	100 (35.7)		
1,000–3,000	37 (32.7)	97 (34.6)		
3,000–5,000	53 (46.9)	33 (11.8)		
>5,000	19 (16.8)	30 (10.7)		
Educational level, n (%)			3.78	0.437
Illiteracy	0 (0.0)	2 (0.7)		
Primary school	23 (20.4)	93 (33.2)		
Junior high school	36 (31.9)	85 (30.4)		
Senior high school	50 (44.2)	96 (34.3)		
Higher than high school	4 (3.5)	4 (1.4)		
Smoking, n (%)			8.30	0.004
Yes	15 (13.3)	83 (29.6)		
No	98 (86.7)	197 (70.4)		
Alcohol consumption, n (%)			42.10	<0.001
Yes	85 (75.2)	115 (41.1)		
No	28 (24.8)	165 (58.9)		
Regular physical exercise, n (%)			4.40	0.036
Yes	60 (53.1)	108 (38.6)		
No	53 (46.9)	172 (61.4)		
BMI category (kg/m^2^), n (%)			85.40	<0.001
<18.5	8 (7.1)	39 (13.9)		
18.5–23.9	67 (59.3)	45 (16.1)		
24.0–27.9	11 (9.7)	59 (21.1)		
28.0–29.9	10 (8.8)	75 (26.8)		
30.0–34.9	17 (15.0)	62 (22.1)		
Physical frailty, n (%)			26.30	<0.001
No	85 (75.2)	113 (40.4)		
Yes	28 (24.8)	167 (59.6)		
Sleep disturbance, n (%)			18.05	<0.001
No	84 (74.3)	154 (55.0)		
Yes	29 (25.7)	126 (45.0)		
Attitude toward aging, n (%)			78.62	<0.001
Positive	84 (74.3)	59 (21.1)		
Negative	29 (25.7)	221 (78.9)		
Number of chronic diseases, n (%)			20.18	<0.001
1	60 (53.1)	82 (29.3)		
2	40 (35.4)	118 (42.1)		
≥3	13 (11.5)	80 (28.6)		
Diabetes mellitus, n (%)			9.81	0.002
Yes	25 (22.1)	111 (39.6)		
No	88 (77.9)	169 (60.4)		

Values are n (%). Chi-square or Fisher’s exact tests as appropriate. BMI, body mass index.

### Distribution of chronic diseases

The most prevalent chronic diseases were hypertension (62.6%), type 2 diabetes mellitus (34.6%), osteoarthritis/osteoporosis (29.3%), coronary heart disease (23.4%), cerebrovascular disease (12.5%), chronic respiratory disease (10.2%), chronic kidney disease (4.6%), and malignant tumor (3.3%). Multimorbidity was common: 142 participants (36.1%) had one chronic disease, 158 (40.2%) had two, and 93 (23.7%) had three or more. The proportion of participants with ≥3 chronic diseases was substantially higher in the oral-frailty group than in the non-oral-frailty group (28.6% vs. 11.5%; *p* < 0.001), pointing to a dose–response relationship between multimorbidity and oral frailty. Disease-by-disease comparison showed that diabetes mellitus (39.5% vs. 22.1%, *p* = 0.002) and cerebrovascular disease (14.6% vs. 6.2%, *p* = 0.026) were more common in participants with oral frailty (see Table 2).

**Table 2:** Distribution of specific chronic diseases by oral-frailty status (*n* = 393).

Chronic disease	All (*N* = 393)	No oral frailty (*n* = 113)	Oral frailty (*n* = 280)
Hypertension	246 (62.6)	68 (60.2)	178 (63.6)
Type 2 diabetes mellitus	136 (34.6)	25 (22.1)	111 (39.6)
Osteoarthritis / osteoporosis	115 (29.3)	30 (26.5)	85 (30.4)
Coronary heart disease	92 (23.4)	23 (20.4)	69 (24.6)
Cerebrovascular disease	49 (12.5)	7 (6.2)	42 (15.0)
Chronic respiratory disease	40 (10.2)	8 (7.1)	32 (11.4)
Chronic kidney disease	18 (4.6)	3 (2.7)	15 (5.4)
Malignant tumor	13 (3.3)	2 (1.8)	11 (3.9)
Others*	57 (14.5)	14 (12.4)	43 (15.4)

Values are n (%). Categories are not mutually exclusive; many participants had ≥2 conditions. *Others include thyroid disease, gout, peptic ulcer, anemia, etc.

### Prevalence and distribution of oral frailty

Oral frailty (OFI-8 ≥ 4) was detected in 280 of 393 participants (prevalence 71.2%). The mean OFI-8 score was 5.61 ± 2.58. At the item level, the most frequently endorsed items were difficulty eating hard food (62.3%), choking on tea/soup (48.6%), dry mouth (45.8%), fewer than 20 natural teeth (38.4%), and difficulty articulating clearly (32.1%). A total of 213 participants (54.2%) reported visiting a dentist less than once per year. In the sensitivity analysis using the more conservative cut-off of ≥5, the prevalence was 53.4%, and the direction and statistical significance of all multivariable associations were essentially unchanged.

### Between-group comparison of characteristics by oral-frailty status

Between-group comparisons (Table 1) showed that age, sex, ethnicity, personal monthly income, BMI category, smoking, alcohol consumption, regular physical exercise, physical frailty, sleep disturbance, attitude toward aging, number of chronic diseases and diabetes mellitus were associated with oral frailty (*p* < 0.10 for all; Tables 3, 4).

**Table 3:** Coding scheme for variables entered into the binary logistic regression model.

Variable	Coding
Sex	Male = 0 (Ref.); Female = 1
Age group (years)	60–69 = 0 (Ref.); 70–79 = 1; ≥80 = 2
Ethnicity	Han Chinese = 0 (Ref.); Korean Chinese = 1
Personal monthly income (RMB)	>5,000 = 0 (Ref.); 3,000–5,000 = 1; 1,000–3,000 = 2; <1,000 = 3; None = 4
Educational level	Senior high school and above = 0 (Ref.); Junior high school = 1; Primary school or below = 2
Smoking	No = 0 (Ref.); Yes = 1
Alcohol consumption	No = 0 (Ref.); Yes = 1
Regular physical exercise	Yes = 0 (Ref.); No = 1
BMI category (kg/m^2^)	18.5–23.9 = 0 (Ref.); <18.5 = 1; 24.0–27.9 = 2; 28.0–29.9 = 3; 30.0–34.9 = 4
Physical frailty	No = 0 (Ref.); Yes = 1
Sleep disturbance	No = 0 (Ref.); Yes = 1
Attitude toward aging	Positive = 0 (Ref.); Negative = 1
Number of chronic diseases	1 = 0 (Ref.); 2 = 1; ≥3 = 2
Diabetes mellitus	No = 0 (Ref.); Yes = 1

**Table 4:** Binary logistic regression results for factors independently associated with oral frailty (*n* = 393).

Variable	β	SE	Wald χ^2^	*p*	Adjusted OR	95% *CI*
Constant	−1.04	1.51	0.47	0.491	0.35	—
Sex (Female vs. Male)	0.93	0.38	5.97	0.015	2.52	1.20–5.31
Age group (Ref. 60–69)						
70–79	0.86	0.42	4.20	0.040	2.36	1.04–5.37
≥80	1.63	0.63	6.71	0.010	5.12	1.51–17.40
Ethnicity (Korean Chinese vs. Han)	−0.72	0.45	2.55	0.110	0.49	0.20–1.18
Personal monthly income (Ref. >5,000 RMB)						
3,000–5,000	0.71	0.49	2.12	0.146	2.04	0.78–5.30
1,000–3,000	1.32	0.55	5.79	0.016	3.75	1.28–11.02
<1,000	1.46	0.50	8.51	0.004	4.30	1.62–11.40
None	1.51	0.69	4.83	0.028	4.54	1.18–17.50
Educational level (Ref. Senior high+)						
Junior high school	0.21	0.32	0.43	0.512	1.23	0.66–2.30
Primary school or below	0.55	0.34	2.66	0.103	1.74	0.89–3.39
Smoking (Yes vs. No)	−1.07	0.48	5.05	0.025	0.34	0.13–0.88
Alcohol consumption (Yes vs. No)	2.10	0.46	20.51	<0.001	8.14	3.29–20.19
Regular physical exercise (No vs. Yes)	0.39	0.42	0.88	0.349	1.48	0.65–3.37
BMI category (Ref. 18.5–23.9)						
<18.5	1.09	0.54	4.12	0.042	2.97	1.04–8.49
24.0–27.9	1.61	0.59	7.53	0.006	5.01	1.59–15.83
28.0–29.9	2.71	0.74	13.38	<0.001	15.05	3.52–64.31
30.0–34.9	2.59	0.62	17.62	<0.001	13.30	3.97–44.51
Physical frailty (Yes vs. No)	0.91	0.45	4.00	0.045	2.48	1.02–6.03
Sleep disturbance (Yes vs. No)	1.20	0.46	6.88	0.009	3.31	1.35–8.11
Attitude toward aging (Negative vs. Positive)	2.61	0.46	32.68	<0.001	13.56	5.55–33.15
Number of chronic diseases (Ref. 1)						
2	0.41	0.31	1.78	0.182	1.51	0.83–2.76
≥3	1.03	0.34	9.11	0.003	2.81	1.43–5.51
Diabetes mellitus (Yes vs. No)	0.74	0.29	6.43	0.011	2.10	1.19–3.71

Adjusted odds ratios derived from a single binary logistic regression including all variables shown. Omnibus χ^2^ = 187.6, df = 14, *p* < 0.001. Hosmer–Lemeshow χ^2^ = 9.21, df = 8, *p* = 0.325. Nagelkerke R^2^ = 0.481. All variance inflation factors were < 2.5. CI, confidence interval; OR, odds ratio; SE, standard error.

## Discussion

In this cross-sectional study of community-dwelling older adults with chronic diseases living in minority communities of Northeast China, oral frailty was highly prevalent (71.2%) and was independently associated with a broad set of demographic, socioeconomic, behavioral, physical, psychosocial and—most importantly—disease-related factors. To our knowledge, this is one of the first studies to explicitly evaluate the contribution of multimorbidity and of diabetes mellitus to oral-frailty risk in this specific population.

Prevalence in international context. The prevalence of 71.2% observed here exceeds that reported in mainland Chinese general community samples (Wang Lin et al., 59.2%; Tang Ji et al., 44.7%) and is similar to figures from Taiwan (60.4%), Yin et al.’s multicentre Chinese study (60.5%), India (67.2%) and Korea (73.0%); it is markedly higher than Japanese community-based estimates (8.4–54.3%) (39–46). Two features of our sample help explain the higher prevalence: first, we exclusively recruited participants with chronic disease, a group with poorer overall health, greater inflammatory burden and frequent polypharmacy; second, our setting is a minority ethnic region with lower educational attainment and socioeconomic status, and traditional dietary patterns rich in fermented, high-salt foods that may compromise oral health. By contrast, Japan combines a mature publicly-funded dental insurance system (>75,000 dental institutions and >160,000 dentists for 127 million people) with widespread preventive dental visits, whereas in our sample only 45.8% reported any dental visit in the previous year. These structural differences likely contribute to the cross-national gradient.

Number of Chronic Diseases and Oral Frailty. Compared with participants who had only one chronic disease, those with three or more chronic diseases exhibited nearly threefold higher adjusted odds of oral frailty (aOR 2.81, 95% CI 1.43–5.51). Several mechanisms plausibly underlie this association: (i) chronic low-grade systemic inflammation accompanying multimorbidity drives both sarcopenia and periodontal destruction; (ii) polypharmacy induces xerostomia—more than 500 prescription medications list dry mouth as a side effect—which compromises oral self-cleansing and increases the risk of caries; (iii) functional decline associated with multimorbidity reduces self-care capacity and adherence to oral hygiene practices; and (iv) nutritional compromise, common in multimorbid older adults, further weakens orofacial musculature. These findings reinforce the call—consistent with the recent systematic review by Wang et al.—to integrate routine oral frailty screening into multimorbidity management protocols (25).

Diabetes mellitus was independently associated with oral frailty (adjusted odds ratio [aOR] 2.10, 95% confidence interval [CI] 1.19–3.71). This finding aligns with a growing body of evidence demonstrating that diabetes is a direct biological driver of oral pathology through multiple well-characterized pathways. Hyperglycemia increases salivary glucose concentrations and shifts the oral microbiome toward periodontopathogens; advanced glycation end-products accumulate in periodontal tissues, accelerating bone resorption; microvascular insufficiency and immune dysregulation impair gingival wound healing; and many anti-diabetic and antihypertensive drugs commonly co-prescribed with anti-diabetic medications cause xerostomia. A recent meta-analysis by Wang et al. (2025) reported a pooled oral frailty prevalence of approximately 65% among older adults with diabetes—substantially higher than in non-diabetic older populations—with the duration of diabetes, glycemic control (HbA1c), and the number of co-prescribed medications emerging as key modifiable determinants (25, 47). Our findings extend this evidence to a Chinese minority community sample and underscore the need for diabetes-specific oral health promotion strategies in primary care diabetes clinics, including routine OFI-8 screening, structured oral hygiene counseling, and timely referral for periodontal care.

Age and sex. Consistent with prior reports, oral frailty increased with age (aOR 5.12 for ≥80 vs. 60–69 years) and was more common in women (aOR 2.52). Physiological aging is accompanied by reduced salivary secretion, gingival recession, root exposure and dental-pulp degeneration, all of which weaken oral self-cleansing and local immunity. The female preponderance is consistent with findings from Hironaka, Nagatani and Kang and may be explained by earlier permanent-tooth eruption, postmenopausal estrogen decline (accelerating alveolar-bone resorption, increasing vascular permeability, reducing salivary flow) and longer life expectancy with cumulative oral wear (48–50). Socioeconomic factors. Personal monthly income showed a strong gradient: participants with no income or <1,000 RMB had more than fourfold higher adjusted odds of oral frailty than those earning >5,000 RMB. Older adults with chronic disease face substantial out-of-pocket medical expenses that crowd out preventive dental care and quality oral-hygiene products. Targeted oral-health subsidies for low-income chronically-ill older adults should be considered. Nutritional status (BMI). Both underweight (<18.5 kg/m^2^) and high BMI (28.0–29.9 kg/m^2^) categories were independently associated with oral frailty, reflecting the dual contribution of malnutrition (weakening type-II muscle fibers in swallowing musculature) and obesity-driven chronic inflammation (enhancing periodontal destruction through adipokine and oxidative-stress pathways) (14, 51). Physical frailty, sleep disturbance, alcohol and smoking. Each of these modifiable factors was independently associated with oral frailty. Physical frailty reflects reduced overall physiological reserve including orofacial musculature; sleep disturbance contributes to systemic inflammation and impaired self-care; alcohol disrupts oral microbial balance and bone-cementum formation (52–54); and smoking alters salivary composition, promotes cariogenic flora, and is a major cause of oral leukoplakia (55, 56). Combined with medication-induced xerostomia common in older adults with chronic disease, these factors substantially elevate risk. Community-based smoking cessation, alcohol-reduction and sleep-hygiene interventions should therefore be incorporated into oral-frailty prevention programs. Attitude toward aging. Negative attitudes toward aging had the strongest single association (aOR 13.56), consistent with the Theory of Planned Behavior, in which attitudes shape behavioral intentions and ultimately health behaviors. Negative perceptions of aging are linked to loneliness, dependency, and reduced self-efficacy that translate into neglect of oral hygiene and dental care (57–59). Psychosocial interventions targeting aging attitudes—group education, peer support, narrative therapy—may therefore yield benefits well beyond oral health.

Implications for community-based practice. From a policy perspective, the findings argue strongly for integrating oral-frailty screening with the OFI-8 into routine community-based chronic-disease management, particularly for diabetic and multimorbid older adults. Marino et al. demonstrated that community-health-worker-delivered oral-health programs are 82% more cost-effective than chairside dentist-delivered education, lending support to a community-centric model (60). Standardized pathways combining oral-frailty screening, oral-hygiene counseling, swallowing-muscle and tongue exercises, smoking-cessation and alcohol-reduction support, attitude-toward-aging interventions, and timely referral to dental services are likely to be needed to address the heterogeneous determinants identified in this study.

## Limitations

This study has several limitations. First, the cross-sectional design precludes causal inference; longitudinal studies are needed to clarify the temporal pathways between chronic diseases and oral frailty. Second, although we included the number of chronic diseases and diabetes mellitus, the small number of participants with individual conditions (e.g., chronic kidney disease, malignancy) precluded fully disease-specific multivariable analyses. Larger studies should perform stratified analyses by disease type and severity. Third, detailed nutritional status (e.g., MNA-SF) and polypharmacy burden data were not available and could not be incorporated. Fourth, the OFI-8 is a screening tool based on self-report; confirmatory objective measures (tongue pressure, occlusal force, oral diadochokinesis) were beyond the resources of this community survey. Future research should combine self-report screening with objective assessments. Fifth, the four-natural-teeth eligibility criterion means our findings do not generalize to edentulous older adults, who would require alternative screening instruments. Finally, the single-region convenience sample limits generalizability beyond minority communities in Northeast China; multi-center studies across diverse Chinese regions are warranted.

## Conclusion

Oral frailty is highly prevalent (71.2%) among community-dwelling older adults with chronic diseases in minority communities of Northeast China. Beyond previously identified demographic, behavioral and psychosocial determinants, multimorbidity (≥3 chronic diseases) and diabetes mellitus emerged as strong, independent risk factors. The findings support the integration of oral-frailty screening into routine community-based chronic-disease management and the development of targeted prevention strategies that bridge medical, dental and public-health services.

## Data Availability

The raw data supporting the conclusions of this article will be made available by the authors, without undue reservation.

## References

[1] World Health Organization. Ageing and Health. Geneva: World Health Organization. (2018).

[2] United Nations, Department of Economic and Social Affairs, Population Division, World Population Prospects 2022: Summary of Results. New York, NY: United Nations (2022).

[3] GBD 2021 Causes of Death Collaborators. Global burden of 288 causes of death and life expectancy decomposition in 204 countries and territories, 1990–2021. Lancet. (2024) 403:2100–32. doi: 10.1016/S0140-6736(24)00367-2 PMC11126520 38582094

[4] Skou T Mair FS Fortin M. Multimorbidity. Nat Rev Dis Primers. (2022) 8:48. doi: 10.1038/s41572-022-00376-4, 35835758 PMC7613517

[5] Su B Li D Xie J Wang Y Wu X Li J . Chronic disease in China: geographic and socioeconomic determinants among persons aged 60 and older. J Am Med Dir Assoc. (2023) 24:206–212.e5. doi: 10.1016/j.jamda.2022.10.002, 36370750

[6] Marengoni A Angleman S Melis R Mangialasche F Karp A Garmen A . Aging with multimorbidity: a systematic review of the literature. Ageing Res Rev. (2011) 10:430–9. doi: 10.1016/j.arr.2011.03.003, 21402176

[7] Tanaka T Takahashi K Hirano H Kikutani T Watanabe Y Ohara Y . Oral frailty as a risk factor for physical frailty and mortality in community-dwelling elderly. J Gerontol A Biol Sci Med Sci. (2018) 73:1661–7. doi: 10.1093/gerona/glx225, 29161342

[8] Parisius KGH Wartewig E Schoonmade LJ Aarab G Gobbens R Lobbezoo F. Oral frailty dissected and conceptualized: a scoping review. Arch Gerontol Geriatr. (2022) 100:104653. doi: 10.1016/j.archger.2022.104653, 35176531

[9] Tanaka T Hirano H Ohara Y Nishimoto M Iijima K. Oral frailty Index-8 in the risk assessment of new-onset oral frailty and functional disability among community-dwelling older adults. Arch Gerontol Geriatr. (2021) 94:104340. doi: 10.1016/j.archger.2021.104340, 33529863

[10] Yang C Gao Y An R Lan Y Yang Y Wan Q. Oral frailty: a concept analysis. J Adv Nurs. (2024) 80:3134–45. doi: 10.1111/jan.16042, 38214108

[11] Minakuchi S Tsuga K Ikebe K Ueda T Tamura F Nagao K . Oral hypofunction in the older population: position paper of the Japanese Society of Gerodontology. Gerodontology. (2018) 35:317–24. doi: 10.1111/ger.12347, 29882364

[12] Watanabe Y Okada K Kondo M Matsushita T Nakazawa S Yamazaki Y. Oral health for achieving longevity. Geriatr Gerontol Int. (2020) 20:526–38. doi: 10.1111/ggi.13921 32307825

[13] Iwasaki M Motokawa K Watanabe Y Shirobe M Inagaki H Edahiro A . Association between oral frailty and nutritional status among community-dwelling older adults: the Takashimadaira study. J Nutr Health Aging. (2020) 24:1003–10. doi: 10.1007/s12603-020-1511-4, 33155629 PMC12280575

[14] Hernandez SG Feldman S Perez-Abalo M. Malnutrition, dysphagia, sarcopenia and weakness in the older population. Dysphagia. (2024) 39:514–21. doi: 10.1007/s00455-023-10636-4, 38078983

[15] Hironaka S Kugimiya Y Watanabe Y Motokawa K Hirano H Kawai H . Association between oral, social, and physical frailty in community-dwelling older adults. Arch Gerontol Geriatr. (2020) 89:104105. doi: 10.1016/j.archger.2020.104105, 32480111

[16] Dibello V Lozupone M Manfredini D Dibello A Zupo R Sardone R . Oral frailty and neurodegeneration in Alzheimer's disease. Neural Regen Res. (2021) 16:2149–53. doi: 10.4103/1673-5374.310672, 33818486 PMC8354109

[17] Ohara Y Iwasaki M Shirobe M Kawai H Edahiro A Motokawa K . Xerostomia as a key predictor of physical frailty among community-dwelling older adults in Japan. Arch Gerontol Geriatr. (2022) 99:104608. doi: 10.1016/j.archger.2021.104608, 34922245

[18] Soto AP Meyer SL. Oral implications of polypharmacy in older adults. Dent Clin N Am. (2021) 65:323–43. doi: 10.1016/j.cden.2020.11.007, 33641756

[19] Kudiyirickal MG Pappachan JM. Diabetes mellitus and oral health. Endocrine. (2015) 49:27–34. doi: 10.1007/s12020-014-0496-3, 25487035

[20] Stoopler ET Villa A Bindakhil M Díaz DLO Sollecito TP. Common Oral conditions: a review. JAMA. (2024) 331:1045–54. doi: 10.1001/jama.2024.0953 38530258

[21] Genco RJ Borgnakke WS. Diabetes as a potential risk for periodontitis: association studies. Periodontol. (2000) 83:40–5. 10.1111/prd.12270 32385881

[22] Preshaw PM Bissett SM. Periodontitis and diabetes. Br Dent J. (2019) 227:577–84. doi: 10.1038/s41415-019-0794-5 31605062

[23] Yu SY Lee SK Yang B. Disparities in preventive oral healthcare and periodontal health among adults with diabetes. Prev Chronic Dis. (2021) 18:E59. doi: 10.5888/pcd18.200594, 33988497 PMC8139443

[24] Ishii M Yamaguchi Y Hamaya H Iwata Y Takada K Ogawa S . Influence of oral health on frailty in patients with type 2 diabetes aged 75 years or older. BMC Geriatr. (2022) 22:145. doi: 10.1186/s12877-022-02841-x, 35183107 PMC8858474

[25] Wang R. Prevalence and influencing factors of oral frailty in older adults with diabetes: a systematic review and meta-analysis. Front Public Health. (2025) 13:1702509. doi: 10.3389/fpubh.2025.1702509, 41393040 PMC12698406

[26] Chen JH Chen Y Chen MM. Regional difference of China's economic development level. J Quant Technical Econ. (2020) 12:108–26.

[27] von Elm E Altman DG Egger M Pocock SJ Gøtzsche PC Vandenbroucke JP. The strengthening the reporting of observational studies in epidemiology (STROBE) statement. Lancet. (2007) 370:1453–7. doi: 10.1016/S0140-6736(07)61602-X 18064739

[28] Hiltunen K Saarela RKT Kautiainen H Roitto HM Pitkälä KH Mäntylä P. Relationship between Fried's frailty phenotype and oral frailty in long-term care residents. Age Ageing. (2021) 50:2133–9. doi: 10.1093/ageing/afab177, 34473831 PMC8581380

[29] Xue W. Statistical Analysis and Application of SPSS. 5th ed. Beijing: China Renmin University Press (2017).

[30] Zhou BF. Cooperative Meta-analysis Group of the Working Group on obesity in China. Predictive values of body mass index and waist circumference for risk factors of certain related diseases in Chinese adults. Biomed Environ Sci. (2002) 15:83–96. 12046553

[31] World Health Organization. International Classification of Diseases, 10th Revision (ICD-10). Geneva: World Health Organization. (1992).

[32] Lin H-Y Yang Y-O Liao C-Y. Establishment of a mandarin Chinese version of the Oral frailty Index-8 and exploration of the association between oral frailty and sarcopenia. J Multidiscip Healthc. (2025) 18:1421–32. 10.3390/geriatrics10020047 PMC11932261 40126297

[33] Yin Y Zhao Y Fei Y Liu Y Ji Y Shan E . Epidemiology and risk factors of oral frailty among older people: an observational study from China. BMC Oral Health. (2024) 24:368. doi: 10.1186/s12903-024-04149-1, 38515048 PMC10958975

[34] Corrêa L Júdice A Scoz R Machado V Mendes JJ Proença L . Portuguese version of the Oral frailty Index-8: instrument validation study. Interact J Med Res. (2024) 13:e49975. doi: 10.2196/49975 PMC11555463 39466299

[35] Kang JH Park SC Jung HI Jung SJ Park HJ Kim SM . Validation of the Korean academy of geriatric dentistry screening questionnaire and oral frailty diagnostic criteria in community-dwelling older adults. Epidemiol Health. (2024) 46:e2024008. doi: 10.4178/epih.e2024008, 38186249 PMC11099569

[36] Wei Y Cao YP Yang XL. Sinicization and reliability and validity of frailty risk screening tool in elderly hospitalized patients. Chin J Pract Nurs. (2018) 34:1526–30.

[37] Liu XC Tang MQ Hu L. Reliability and validity of Pittsburgh sleep quality index. Chin J Psychiatry. (1996) 2:103–7.

[38] Huang YF Wang DH Liu YG. A preliminary trial of the Chinese version of the aging attitude questionnaire (AAQ). Chin J Clin Psychol. (2010) 18:447–50.

[39] Lin W Mei J Ting W. Analysis of the current status and influencing factors of oral frailty risk in the elderly population in the community. J Nurs (China). (2023) 38:112–6.

[40] Tang J Tang XY Zeng L. Analysis of the current situation and influencing factors of oral weakness in the elderly population in rural areas of Guizhou Province. Chin J Chronic Dis Prev Control. (2023) 31:327–31.

[41] Kuo YW Lee JD. Association between oral frailty and physical frailty among rural middle-old community-dwelling people with cognitive decline in Taiwan. Int J Environ Res Public Health. (2022) 19:2884. doi: 10.3390/ijerph19052884, 35270577 PMC8909940

[42] Ayoob A Janakiram C. Prevalence of physical and oral frailty in geriatric patients in Kerala. India J Oral Biol Craniofac Res. (2024) 14:158–63. doi: 10.1016/j.jobcr.2024.01.011, 38347898 PMC10859288

[43] Kugimiya Y Watanabe Y Ueda T Motokawa K Shirobe M Igarashi K . Rate of oral frailty and oral hypofunction in rural community-dwelling older Japanese individuals. Gerodontology. (2020) 37:342–52. doi: 10.1111/ger.12468, 32141117

[44] Li T Shen Y Leng Y Zeng Y Li L Yang Z . The prevalence of oral frailty among older adults: a systematic review and meta-analysis. Eur Geriatr Med. (2024) 15:645–55. doi: 10.1007/s41999-023-00930-7, 38528284

[45] Zhang QY. Research on oral insurance in developed countries — taking the United States and Japan as examples. Modern Commerce and Industry. (2022) 43:97–8.

[46] Nomura Y Ishii Y Suzuki S Morita K Suzuki A Suzuki S . Nutritional status and oral frailty: a community-based study. Nutrients. (2020) 12:2886. doi: 10.3390/nu12092886, 32967313 PMC7551233

[47] Ren W Feng H Cheng J Guo Z Tang Q. Mediating role of nutritional status in the association between oral-frailty risk and HbA1c levels among elderly patients with type 2 diabetes. Int J Biol Life Sci (2025) 12:12–17. doi: 10.54097/mky1x393

[48] Nagatani M Tanaka T Son BK Kawamura J Tagomori J Hirano H . Oral frailty as a risk factor for mild cognitive impairment in community-dwelling older adults: Kashiwa study. Exp Gerontol. (2023) 172:112075. doi: 10.1016/j.exger.2022.112075, 36581224

[49] Pahel BT Vann WF Jr Divaris K Rozier RG. A contemporary examination of first and second permanent molar emergence. J Dent Res. (2017) 96:1115–21. doi: 10.1177/0022034517716395, 28644755 PMC5582687

[50] Eldessouky HF Marie M. Nonsurgical treatment of periodontitis in menopausal patients: a randomized control trial. Biomed Res Int. (2024) 2024:6997142. 10.1155/2024/6997142 PMC10954359 38510979

[51] Randolph ME Luo Q Ho J Vest KE Sokoloff AJ Pavlath GK. Ageing and muscular dystrophy differentially affect murine pharyngeal muscles. J Physiol. (2014) 592:5301–15. doi: 10.1113/jphysiol.2014.280420, 25326455 PMC4262340

[52] Komatsu R Nagai K Hasegawa Y Okuda K Okinaka Y Wada Y . Association between physical frailty subdomains and oral frailty in community-dwelling older adults. Int J Environ Res Public Health. (2021) 18:2931. doi: 10.3390/ijerph18062931, 33809322 PMC8001836

[53] Fan X Peters BA Jacobs EJ Gapstur SM Purdue MP Freedman ND . Drinking alcohol is associated with variation in the human oral microbiome in a large study of American adults. Microbiome. (2018) 6:59. doi: 10.1186/s40168-018-0448-x, 29685174 PMC5914044

[54] Corrêa MG Gomes Campos ML Marques MR Ambrosano GMB Casati MZ Nociti FH Jr . Alcohol intake may impair bone density and new cementum formation. J Periodontal Res. (2016) 51:60–9. doi: 10.1111/jre.12279, 25959998

[55] Yagyuu T Funayama N Imada M Kirita T. Effect of smoking status and PD-L1 expression on the microenvironment and malignant transformation of oral leukoplakia. PLoS One. (2021) 16:e0250359. doi: 10.1371/journal.pone.0250359, 33861793 PMC8051817

[56] Granero Fernandez M Lopez-Jornet P. Association between smoking, glycaemia, blood lipoproteins and risk of oral leukoplakia. Aust Dent J. (2017) 62:47–51. doi: 10.1111/adj.12431, 27219072

[57] Cheng X Cosco TD Ariyo T. Decreasing social isolation to enhance mental health among older adults in China: a mediation analysis of aging attitude. Front Psychol. (2021) 12:735740. doi: 10.3389/fpsyg.2021.735740, 34630248 PMC8499704

[58] Sussman R Gifford R. Causality in the theory of planned behavior. Personal Soc Psychol Bull. (2019) 45:920–33. doi: 10.1177/0146167218801363, 30264655

[59] Ayeh JK Au N Law R. Predicting the intention to use consumer-generated media for travel planning. Tour Manag. (2013) 35:132–43. doi: 10.1016/j.tourman.2012.06.010

[60] Mariño RJ Fajardo J Calache H Morgan M. Cost-minimization analysis of a tailored oral health intervention designed for immigrant older adults. Geriatr Gerontol Int. (2014) 14:336–40. doi: 10.1111/ggi.12103, 23773315

